# The impact of telehealth care on escalation to emergency care: A systematic review and meta-analysis

**DOI:** 10.1177/1357633X241259525

**Published:** 2024-06-05

**Authors:** Anna M Scott, Sharon Sanders, Tiffany Atkins, Madeleen van der Merwe, Carla Sunner, Justin Clark, Paul Glasziou

**Affiliations:** 1 Nuffield Department of Population Health, 6396 University of Oxford, Oxford, UK; 2 Institute for Evidence-Based Healthcare, 3555 Bond University, Robina, Australia; 3 School of Nursing and Midwifery, College of Health, Medicine and Wellbeing, The University of Newcastle, Callaghan, Australia

**Keywords:** Telehealth, telemedicine, emergency care, primary care, aged care, systematic review, meta-analysis

## Abstract

**Objective:**

We compared the impact of accessing healthcare (1) by telehealth (via telephone or video) vs face-to-face; and (2) by telephone vs video telehealth care, on escalation to emergency care.

**Methods:**

We searched Medline, Embase and Cochrane CENTRAL to 24 July 2023; and conducted a citation analysis on 19 September 2023. We included randomised controlled trials. Risk of bias was assessed using Cochrane Tool 2. We calculated risk ratios for dichotomous outcomes and standardised mean difference for continuous outcomes.

**Results:**

Ten trials compared telehealth (five telephone, four video, one both) to face-to-face care. Six were overall low, three some concerns and one high risk of bias. There were no differences between telehealth and face-to-face for visits to the emergency department (RR 1.07, 95% CI 0.89 to 1.29), hospitalisations up to 12 months (RR 0.89, 95% CI 0.56 to 1.41), deaths or other adverse events. Costs of care were similar, as were patient satisfaction scores.

Six trials compared telephone to video telehealth: three were overall low, two some concerns, and one high risk of bias. There were no differences between telephone and video for visits to the emergency department (RR 0.67, 95% CI 0.41 to 1.12), hospitalisations (RR 1.04, 95% CI 0.73 to 1.48), deaths, other adverse events, costs, or patient satisfaction. Healthcare provider satisfaction was high.

**Conclusions:**

Telehealth care – delivered by telephone or by video – may be an appropriate alternative to face-to-face provision of care, as it does not increase the likelihood of escalation of care to the emergency department for patients in primary care, hospital outpatients, post-discharge patients or residents in aged care.

## Background

Telehealth involves the provision of healthcare services remotely, using information and communications technologies, such as video conferencing, teleconferencing, remote monitoring, mobile apps and other technologies; it may be provided synchronously (‘live’) or asynchronously (e.g., through remote monitoring).^1,2^

Prior to 2020, telehealth was available in Australia on a limited basis.^3^ However, with the declaration by the World Health Organisation of the COVID-19 pandemic in March 2020,^4^ the temporary payment of benefits for telehealth was enabled in Australia, allowing for the ongoing provision of telehealth care services by general practitioners, allied healthcare professionals and specialists.^5^ The adoption of telehealth has been considerable. In 2022, there were over 45 million telehealth consultations in Australia. By provider, telehealth consultations represented 22% of all GP consultations, 14% of specialist consultations, 27% of mental health consultations, 28% of nurse practitioner consultations and 15% of allied health consultations.^6^ In December 2021, the Australian Government announced an investment of $106 M to support the permanent implementation of telehealth services as part of the Medicare Benefits Schedule.^7^

Telehealth can increase the accessibility of health care services particularly in areas of provider shortages,^8^ improve healthcare equity,^2^ reduce costs to the healthcare system^9^ and reduce travel time and inconvenience to patients.^10^ Patients are often satisfied or very satisfied with telehealth care^11^ and previous systematic reviews have shown no differences in satisfaction scores between those patients who received care face-to-face and those who received it by telehealth.^12–15^ Evidence also suggests that outcomes for clinical care provided by live telehealth (e.g., by telephone or videoconferencing) are no different to those for face-to-face care, for a broad range of clinical areas and conditions, in primary, allied and specialist care.^16–18^

However, whilst diagnostic accuracy requiring history-taking only is similarly effective for telehealth and face to face care, tele-diagnosis can be challenging when physical examination is necessary,^17,19^ raising the question of whether telehealth may have higher rates of patient transfer to emergency care than face to face care.

To our knowledge, no systematic review has thus far been conducted to answer this question, although a recent scoping review, focusing specifically on residential aged care population, explored the evidence around the impact of the use of telehealth for transfers to emergency care.^20^ We therefore conducted a systematic review with meta-analyses, with a broader focus – i.e., including individuals in both primary and aged care settings – and comparing the effect of live telehealth and face-to-face care, on patient transfer to the emergency department.

## Methods

This systematic review is reported following the Preferred Reporting Items for Systematic Reviews and Meta-Analyses (PRISMA) statement and the review protocol was developed prospectively although was not made publicly available. We followed the ‘2-week systematic review’ (2weekSR) processes for this review.^16,21^

### Inclusion criteria

#### Participants

We included studies conducted in people of any age, gender, or condition, receiving care in primary care and/or in residential aged care (nursing home, residential aged care homes, etc.) setting. We excluded studies in tertiary care (in-hospital patients). Studies conducted in people discharged from hospital and undergoing care by one of the following care providers were included.

Care had to be provided by:

- General Practitioners
- Allied healthcare providers: e.g., psychologist, occupational therapist, physiologist, practice nurse, speech pathologists, Aboriginal and Torres Strait Islander healthcare practitioners and workers, etc.
- Nurse practitioners
- Midwives

We excluded studies in which care was provided by specialists (e.g., psychiatrists, dermatologists, rheumatologists, etc.), unless the care *also* included both the patient and one of the includable healthcare providers (i.e., the care involved, for example, a patient, a GP and a psychiatrist). Solely clinician-to-clinician consultations (i.e., those not involving patients) were excluded.

#### Interventions

We included studies evaluating the effectiveness of real-time (synchronous) consultations via video or telephone. Studies of consultations involving asynchronous provision of care (e.g., store and forward of patient generated data) were excluded.

We excluded studies evaluating mobile apps, virtual reality, texting (e.g., reminders), online based platforms (e.g., information and support systems), telemonitoring and studies of novel (non-standard) interventions.

Consultations could include single or multiple episodes of care, but the compared groups had to receive similar care in terms of frequency, duration and healthcare provider.

#### Comparators

Comparison 1: We included studies comparing consultations via video or telephone, to face-to-face (in-person) consultations.

Comparison 2: We included studies comparing consultations via video to consultations provided by telephone. This addition is a deviation from the protocol, and was added following a request of the Department of Health (Australia) which commissioned this review (see Conflict of Interest/Funding statement).

#### Outcomes (primary, secondary)

The primary outcomes were: visits to the emergency department (Emergency Department, Emergency Room, Accident & Emergency, etc.) and hospital admissions (hospital admittance, hospitalisations, etc.)

The secondary outcomes were: safety (including adverse events, mortality), costs (cost effectiveness, direct costs, etc.), patient satisfaction and healthcare provider satisfaction.

#### Study design

We included randomised controlled trials (RCTs) of any design (parallel, cluster, crossover, factorial or mixed), with more than 10 participants. Systematic reviews were excluded, although where identified, they were searched for additional includable studies. All other study designs (non-randomised trials, observational studies, qualitative-only studies) and all other types of reviews (e.g., literature, scoping, etc.) were excluded.

### Publication type and language

We did not impose restrictions by language (i.e., publications in any language were includable). We included only those publications that were published in full. That is, we excluded publications available as abstract only (e.g., conference abstract) with no additional results information available (e.g., from a clinical trial registry record).

### Search strategies to identify studies

We searched the following databases, from inception to 24 July 2023: Medline (via PubMed), Embase (via Elsevier.com) and CENTRAL via the Cochrane Library (which includes the clinicaltrials.gov and the World Health Organisation's International Clinical Trial Registry Platform, ICTRP). Full search strings are provided in Appendix 1. On 19 September 2023, we performed a backwards and forwards citation analysis on all included studies, using SpiderCite (https://sr-accelerator.com/#/spidercite).

### Study selection and screening

Pairs of review authors (AMS, TA, MvdM, SS, PG) independently screened the titles and abstracts, and full-texts for inclusion. Any disagreements were resolved by discussion, or reference to another author. The selection process was recorded in sufficient detail to complete a PRISMA flow diagram, as well as a list of excluded (full-text) studies with reasons for exclusions (Appendix 2 and 3).

### Data extraction

We used a data extraction form to extract data from each included study. The form was piloted on two studies. Pairs of review authors (AMS, PG, TA) independently extracted the data, and where discrepancies were identified, they were resolved by discussion or by reference to another author. Data were extracted on each study's:

- Study characteristics and methods, including participants, interventions and comparators
- Primary and secondary outcomes prespecified in the ‘outcomes’ section, above
- Data to inform the risk of bias ratings

### Assessment of risk of bias in included studies

Two review authors (AMS, TA) independently assessed the risk of bias for each included study using the RoB 2 tool, as outlined on the *Cochrane Handbook*.^22^ (This is a deviation from the protocol, which pre-specified Cochrane Risk of Bias Tool 1). Cochrane Risk of Bias 2 was used for parallel arm randomised trials, and Cochrane Risk of Bias Tool 2 for cluster-randomised trials was used for cluster trials. Each potential source of bias was graded as low, high or some concerns, with judgements supported by a quote from the relevant trial.

### Measurement of effect and data synthesis

RevMan 5 was used to calculate the treatment effect. We used risk ratios or rate ratios for dichotomous outcomes – risk ratios for results reporting the number of individuals with an event, and rate ratios for the results reporting the number of events only. For continuous outcomes, we used mean difference or standardised mean difference as appropriate.

We undertook meta-analyses when ≥2 studies or comparisons reported the same outcome; anticipating considerable heterogeneity, we used a random effects model. We used the I^2^ statistic to measure heterogeneity among the included trials.

The individual was used as the unit of analysis, where possible. However, where data on the number of individuals with primary and secondary outcomes of interest was not available, we extracted the information as it was presented.

We did not contact investigators or study sponsors to provide missing data.

We planned to assess publication bias using a funnel plot but as there were fewer than 10 trials included in any meta-analysis this was not possible.

### Subgroup and sensitivity analyses

We had intended to conduct subgroup analyses by condition and by timepoint at which the event was measured. Data were not sufficient to conduct subgroup analyses by condition, but we were able to conduct subgroup analyses by timepoint for the hospitalisation outcome. We had intended to conduct a sensitivity analysis by including versus excluding studies at high risk of bias (three or more domains rated at high risk of bias), but none of the included studies had three or more domains rated at high risk of bias.

## Results comparison 1: telehealth (phone or video) vs. Face-to-Face care

### Results of the search

**Figure 1.**
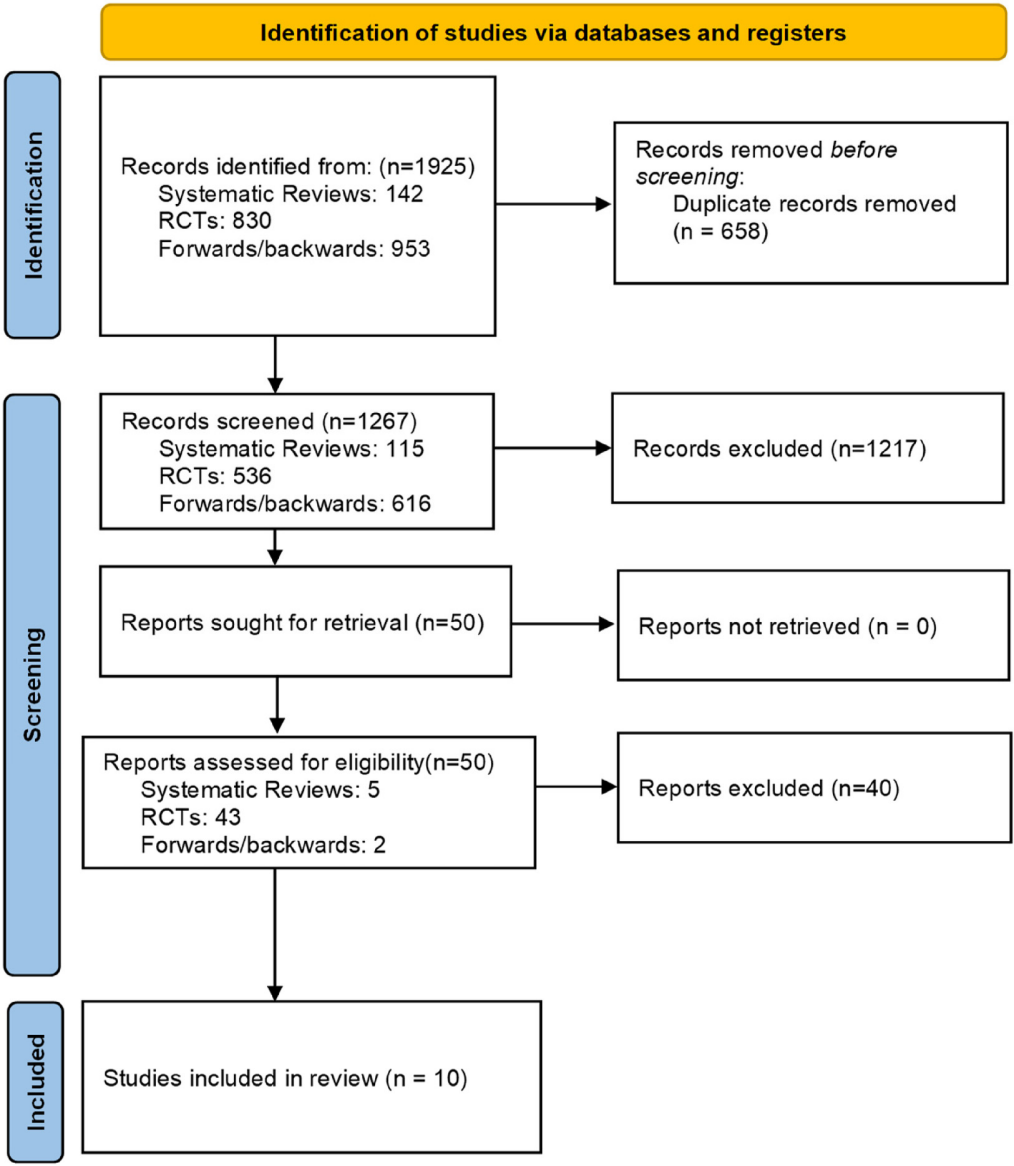
PRISMA flow diagram.

The searches yielded in total 1925 references; 658 duplicates were removed and 1267 references were screened in title and abstract. We excluded 1217 references, and screened 50 references in full-text. We included 10 studies comparing telehealth (by phone or video) to face-to-face provision of care (Figure 1).

### Characteristics of the included studies

All trials were conducted in high-income countries, including the UK (three trials), USA (two trials), Denmark (two trials) and Norway, France and Greece (one trial each). Two trials were cluster and eight were parallel arm trials (six 2-arm trials and two 3-arm trials). Follow-up duration was most commonly for 12 months (five trials), with the remainder lasting up to 90 days (five trials). Participant populations varied, including general practice patients (three trials), nursing home residents (one trial) and patients discharged from the hospital or outpatient care (six trials). The individually randomised trials ranged in size from 103 to 388 participants; the two cluster randomised trial ranged in size from 428 to 20,990 participants in aggregate. Telehealth care was provided either by phone (five trials) or by video (four trials); one trial enabled participants to access care via telephone or video conferencing. Comparators were face-to-face consultations with the healthcare providers (Table 1).

**Table 1.** Characteristics of included studies (comparison 1: telehealth vs face-to-face care).

Author, Year (Country)	RCT Design	Setting	Follow-up duration	Participants	Number of participants randomised N total (N TH group, N F2F group, N other groups if applicable)	Intervention type	Intervention: dose, duration, frequency	Comparator type	Comparator dose, duration, frequency	Sample size calculation (SS)? Powered (P)?
Augestad 2020 Norway^28^	Parallel 2-arm	Hospital Outpatient	12 months post-surgery	Patients with a postsurgical stoma (ileostomy or colostomy)	110 (52 TH, 58 F2F)	F2F baseline + Video consultations	Referred to GP and local stoma nurse who organised baseline (0–3 month), 6, 9, > 12 month consultation	F2F baseline + F2F consultations	Regular stoma follow-up at outpatient clinic baseline, 6, 9, > 12 month consultations	SS: Yes P: Yes
ESTEEM trial Campbell 2015 UK^29^	Cluster	Primary care	28 days	Patients who requested a same day consultation	20,990 (GP TH arm 6695; Nurse TH arm 7012; F2F arm 7283)	Telephone baseline + Telephone consultations	Nurse or GP telephone; would be called by either a GP or nurse later that day to discuss needs.	Telephone baseline + F2F consultations	Followed the standard F2F protocols for that practice	SS: Yes P: Yes
Gayot 2022 France^44^	Cluster	Nursing home	up to 12 months	NH residents > 60 years with multiple chronic diseases	428 (9NHs) (214 (4 NHs) TH, 214 (5NHs) F2F)	F2F baseline + 3 telephone consultations + F2F (12 months)	Initial consults within 10 days (with a geriatrician); 3 follow-up preventative teleconsultations performed at 3, 6, 9 months and lasted 15 to 30 min (with a physician)	F2F baseline + Usual physician care + F2F (12 months)	Usual physician care	SS: Yes P: Yes
Godtfredsen 2020 Denmark^45^	Parallel 2-arm	Hospital outpatient	12 months	Patients with severe COPD	134 (67 TH, 67 F2F)	Baseline F2F + 10 weeks Video + 3 months +12 months	10 weeks physiotherapist-supervised online telerehab programme with a screen provided in home; assessments made baseline, at the end, and 3, and 12 months after end	Baseline F2F + 10 weeks F2F + 3 months + 12 months	10 weeks and assessments made at baseline, at the end, and 3, and 12 months after end of programme	SS: No P: NR
Gruffydd-Jones 2005 UK^26^	Parallel 2-arm	Primary care	12 months	Adult patients with asthma recruited from one semi-rural practice	194 (97 TH, 97 F2F)	Telephone baseline + 2 telephone contacts	Telephone contact at 6 monthly intervals by asthma nurse & action plan	F2F baseline +2 F2F visits	At baseline, 6 &12 months & action plan	SS: Yes P: No
Lindegaard, 2017 Denmark^46^	Parallel 3-arm	Hospital discharged	30 and 90 days after discharge	Malnourished geriatric patients and patients at risk of malnutrition (MNA < 24) and aged 75 and older	208(68 TH, 73 F2F, 67 control)	Standard care in hospital at baseline + 3×Telephone consults	Telephone consult: 15 min -individualised nutritional follow-up after discharge by dietician	Standard care in hospital at baseline + 3×F2F at home	Home visits 3 sessions- lasted 45 min (1, 2, and 4 weeks post discharge	SS: Yes P: Yes
McKinstry 2002 UK^30^	Parallel 2-arm	Primary care	up to 2 weeks for some outcomes	Patients who used the telephone to request same-day appointments from 2 urban practices	388 (194 TH,194 F2F)	1×Telephone	Doctor would phone patients back for telephone appointment and if necessary, would see them later in the day-mean time of appointment 6.7 min (4.9)	1×F2F	Face to face appointment on that day; mean time 8.2 min (4.2)	SS: Yes P: Yes
Pekmezaris, 2012 USA^27^	Parallel 2-arm	Hospital discharged	up to 30 days follow-up (total 90 days observation)	Patients discharged with primary or secondary diagnosis of HF–referred to home care post-hospitalisation >=65 years	168 (83 TH, 85 F2F)	5×F2F nurse visits + Video visits	Live nursing visits and video visits. initial in-person visit for functional classification then 60-day intervention + 30 days of follow-up for a total of 90 days	F2F at home, frequency on nurse's judgement	Live, F2F nursing visits only	SS: Yes P: Yes
Thompson, 2019 USA^47^	Parallel 2-arm	Hospital discharged	up to 3 months	Women scheduled for pelvic surgery	103 (52 TH, 51 F2F)	F2F baseline + 3×Telephone	Phone call by a registered nurse at 2,6, and 12 weeks post operatively	F2F baseline + 3×F2F	Visits at 2, 6, and 12 weeks post-operation	SS: Yes P: Yes
Vasilopoulou 2017 Greece^23^	Parallel 3-arm	Hospital outpatient	12 months but 14 months from baseline	Clinically stable COPD patients attending outpatient clinic	150 (50 TH,50 F2F, 50 usual care)	F2F baseline + 144×Home based telerehab (phone or video)	144 sessions performed over 12 months via phone or video (by a physiotherapist)	F2F baseline + 96×F2F & UC	2x/week for 12 months (96 sessions performed over 12 months)	SS: Yes P: Yes

F2F: face-to-face; UC: usual care; TH: telehealth; RCT: Randomised controlled trial; ED: Emergency department; GP: general practitioner/general practice; RACF: residential aged care facility; NH: nursing home; COPD: chronic obstructive pulmonary disease; HF: heart failure; MNA: Mini Nutritional Assessment; ACE: Aged care emergency service.

### Assessment of risk of bias

#### Risk of bias for parallel arm trials (n = 8)

**Figure 2.**
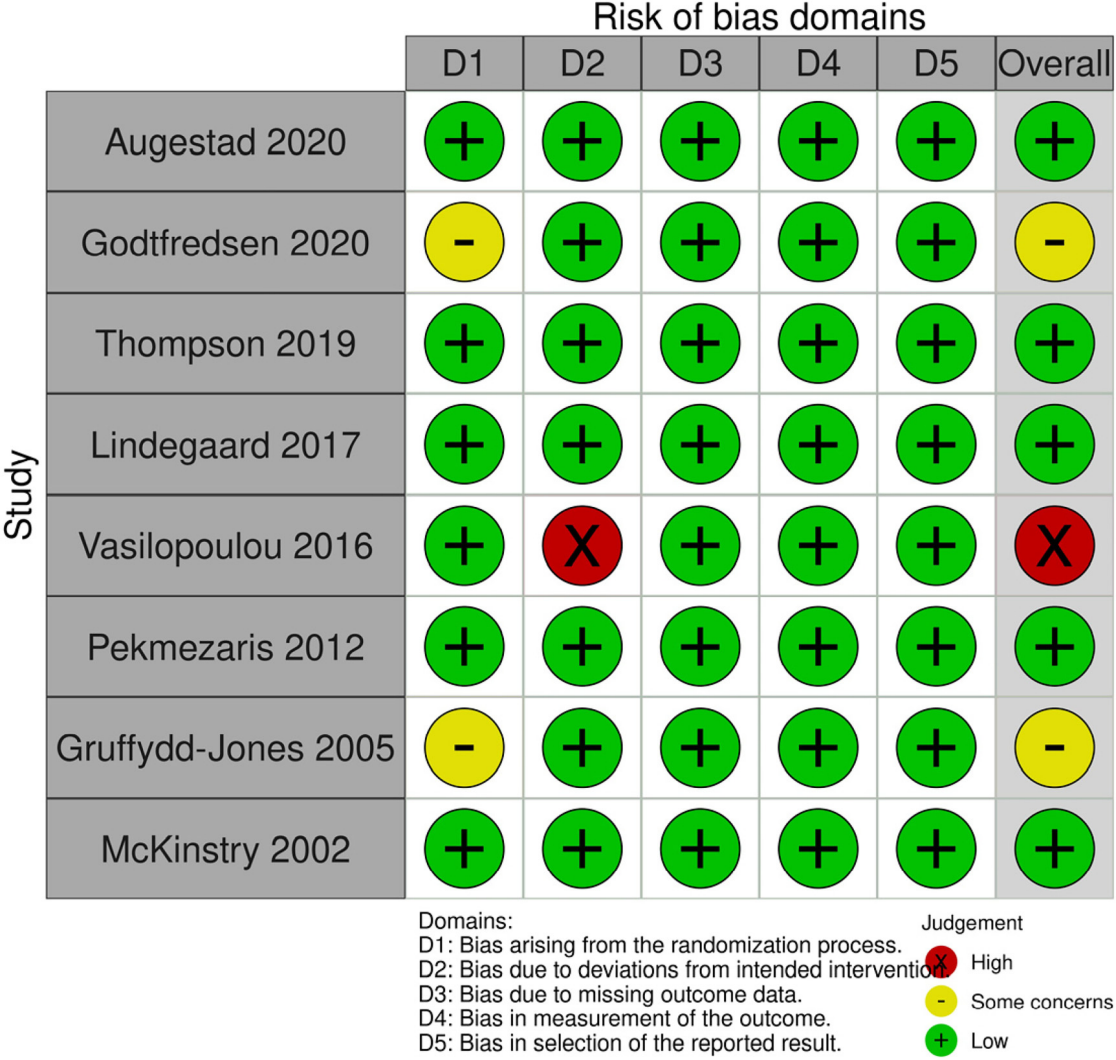
Risk of bias (parallel arm randomised trials).

For parallel arm trials, the risk of bias was generally low or some concerns. Bias due to missing outcome data, bias in measurement of the outcome and bias in the selection of the reported result were low for all studies. Bias due to the randomisation process was low in six of the eight trials (two were rated some concerns). One trial was rated at high risk of bias due to deviations from the intended intervention; remaining trials were rated low risk of bias. Overall, risk of bias was rated as low for five trials, some concerns for two trials, and high for one trial (Figure 2).

#### Risk of bias for cluster randomised trials (n = 2)

**Figure 3.**
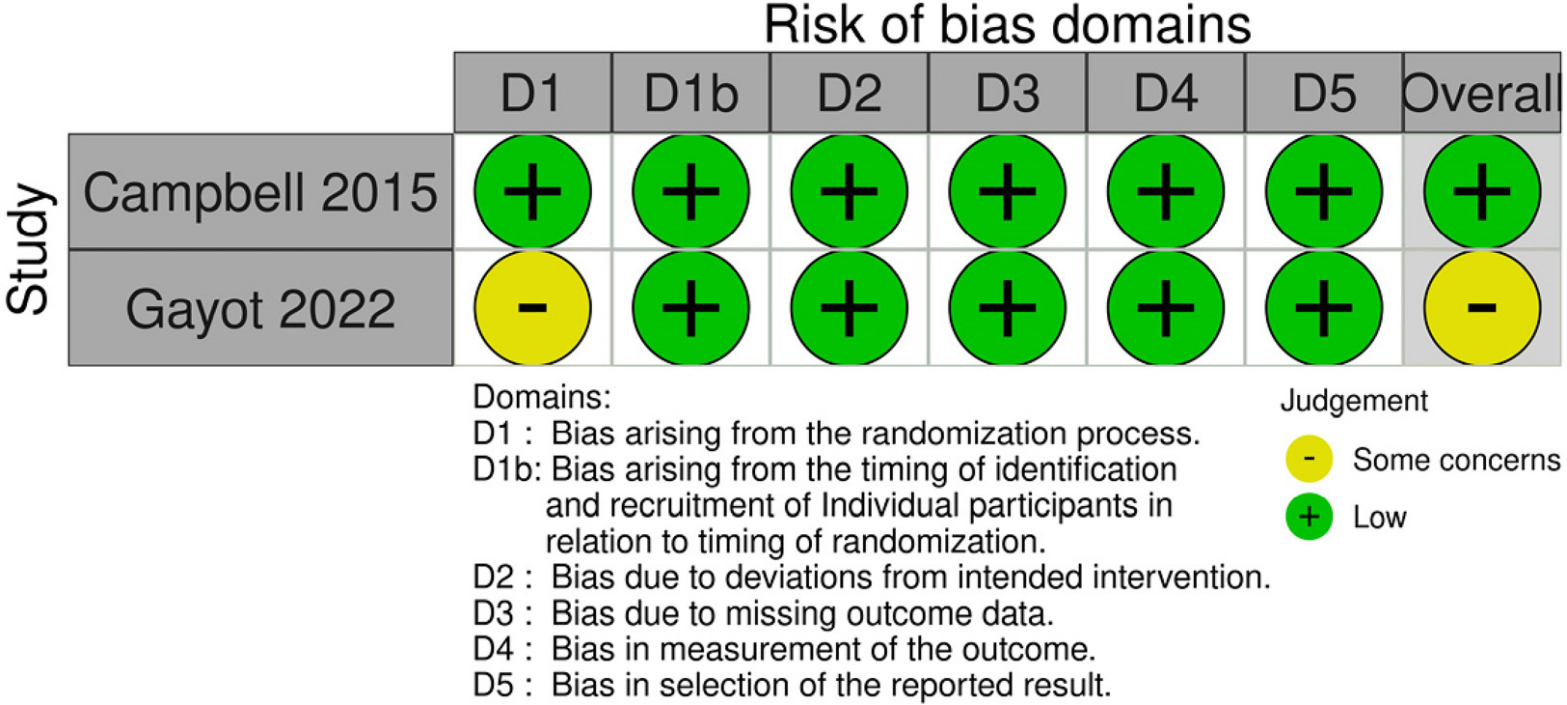
Risk of bias (cluster randomised studies).

Both trials were rated at low risk of bias for timing of the identification and recruitment of individual participants, deviations from the intended intervention, missing outcome data, measurement of the outcome and selection of the reported result. One trial was rated at low risk of bias, and one was rated unclear risk of bias in the randomisation process. Overall, the risk of bias was low for one trial and some concerns for one trial (Figure 3).

### Primary outcome: visits to the emergency department

**Figure 4.**
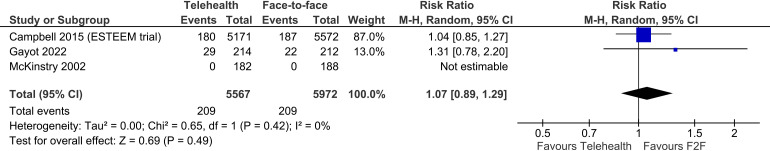
Telehealth vs face-to-face – visits to the emergency department.

Four trials reported on the visits to the emergency department; three were meta-analysable. There was no difference between the telehealth and face-to-face groups for visits to the emergency department (three trials, n = 11,539, RR 1.07, 95% CI 0.89 to 1.29, p = 0.49). Heterogeneity was very low between the studies (I2 = 0%) (Figure 4).

One trial, conducted with clinically stable COPD patients, either attending hospital outpatient clinic (face-to-face) or receiving rehabilitation over video was not meta-analysable.^23^ The video (telerehabilitation) group had a significantly lower rate of visits to the ED in the 12 months of follow-up than the face-to-face group (TH mean 0.5 +/- 0.9 vs F2F mean 1.8 +/- 1.5; p < 0.001).

### Primary outcome: hospital admissions

**Figure 5.**
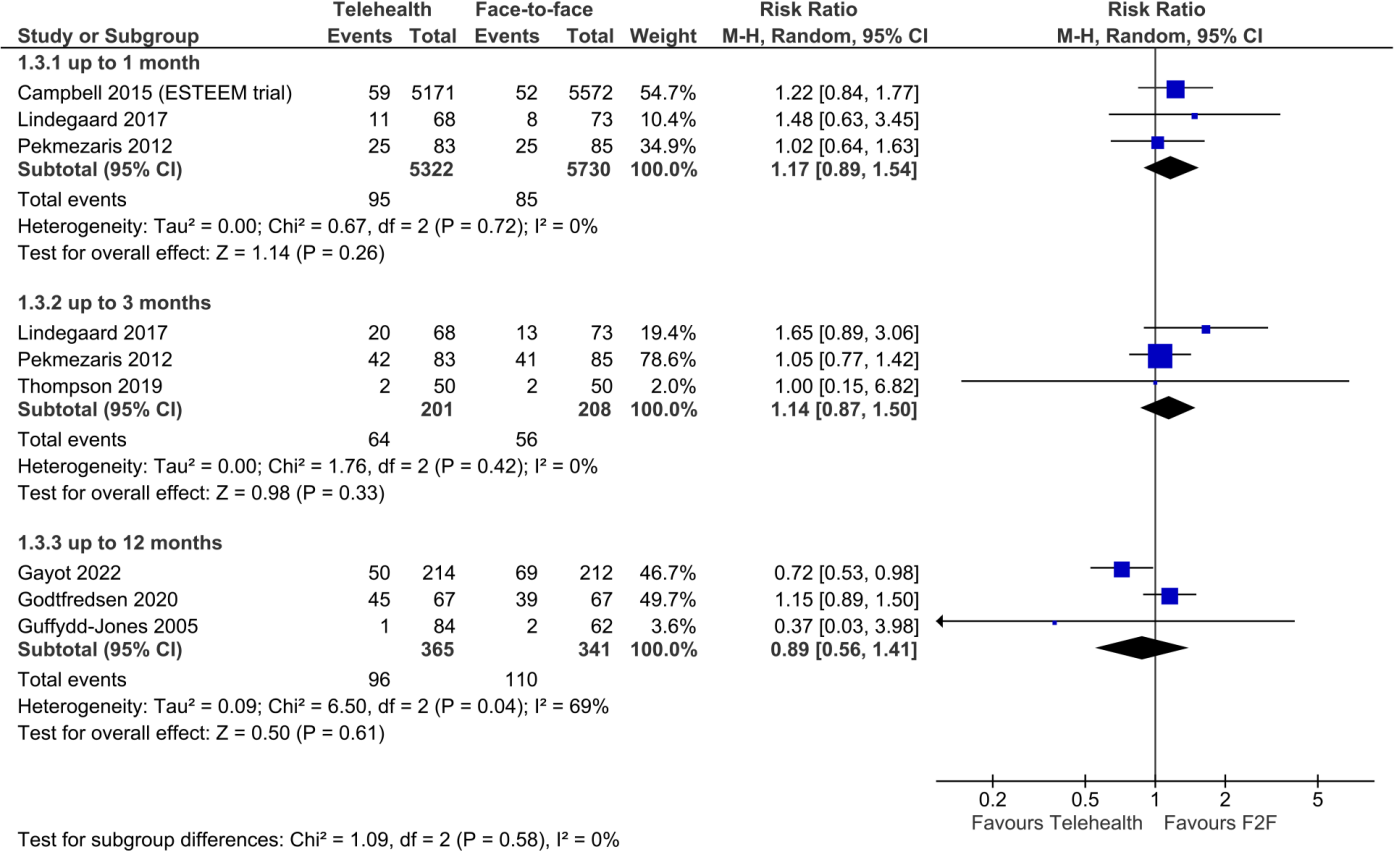
Telehealth vs face-to-face – hospitalisations.

Hospitalisations were reported by eight trials; seven were meta-analysable, and could be subgrouped by timepoint up to which hospitalisations were measured. In trials measuring hospitalisations up to one month post-intervention, there was no difference between telehealth and face-to-face groups (three trials, n = 11,052, RR 1.17, 95% CI 0.89 to 1.54, p = 0.26, I^2 ^= 0%). Three trials measured hospitalisations up to three months, showing no difference between telehealth and face-to-face groups (three trials, n = 409, RR 1.14, 95% CI 0.87 to 1.50, p = 0.33, I^2 ^= 0%). Finally, there was also no difference in hospitalisations between groups up to 12 months (three trials, n = 206, RR 0.89, 95% CI 0.56 to 1.41, p = 0.61), however, heterogeneity between the trials was high (I^2 ^= 69%). (Removing the Gayot study decreases the heterogeneity to 0%, and the difference between groups remains non-significant: RR 1.14, 95% CI 0.88 to 1.48, p = 0.33, I^2^ = 0% – data not shown) (Figure 5).

One non-meta-analysable trial, conducted with clinically stable COPD patients, found that the video and face-to-face rehabilitation groups had very similar rates of hospitalisations for acute exacerbations of COPD: telehealth group (mean 0.3 +/- 0.7) and face-to-face group (mean 0.3 +/- 0.6).^23^

### Secondary outcome: safety (including adverse events, mortality)

#### Deaths

**Figure 6.**
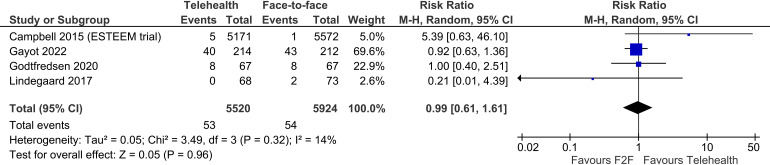
Telehealth vs face-to-face – deaths.

Four trials reported on deaths, showing no difference between telehealth and face-to-face groups in this outcome (four trials, n = 11,444, RR 0.99, 95% CI 0.61 to 1.61, p = 0.96, I2 = 14%) (Figure 6).

#### Adverse events (other than death)

**Figure 7.**
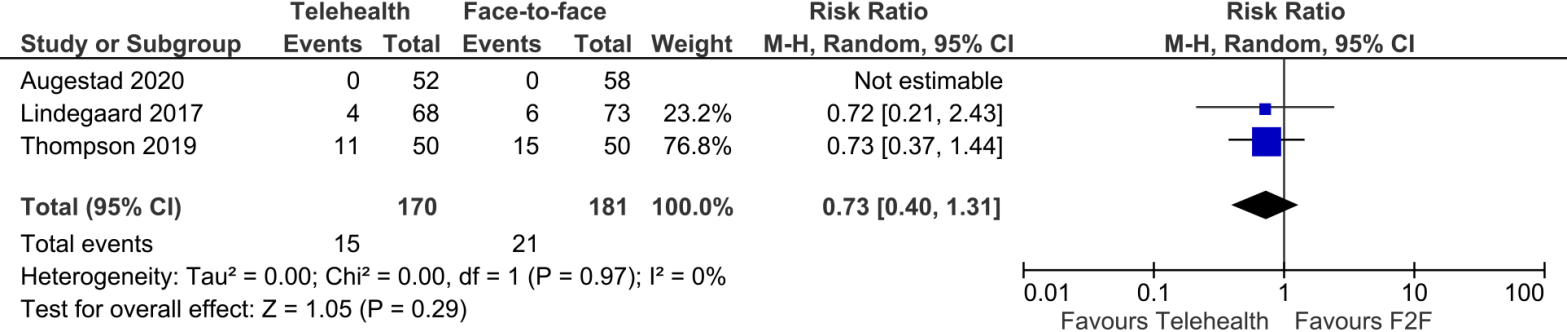
Telehealth vs face-to-face – adverse events (other than death).

Three trials reported on adverse events other than deaths, showing no difference between telehealth and face-to-face groups (three trials, n = 351, RR 0.73, 95% CI 0.40 to 1.31, p = 0.29, I^2 ^= 0%). (Figure 7)

### Secondary outcome: costs

Four trials reported on costs but were not meta-analysable, and are therefore summarised narratively.

Campbell 2014 (the ESTEEM trial)^24^ found very similar costs for telehealth and face-to-face follow-up of primary care patients in the UK. The estimated 28 day follow-up mean costs per patients was 75.41 GBP for face-to-face and 75.21 GBP for the telehealth follow-up.

Gayot (2022)^25^ was based in France, with nursing home residents with multiple chronic diseases. The cost differences were not significant from the perspective of health insurance (telehealth mean and SD: $1900 +/- $3040, face-to-face: $2250 +/- $3450, p = 0.274); or from the perspective of the care provider (telehealth mean and SD: $2290 +/- $4600, face-to-face $2470 +/- $4120, p = 0.662).

Gruffydd-Jones (2005)^26^ reported on mean National Health Service (NHS) costs of care for patients with asthma in the primary setting in the UK. The difference between telehealth group (mean NHS cost 210 GBP/patient/year) and face-to-face group (mean NHS cost 334 GBP/patient/year) was not significant (p = 0.071).

Pekmezaris (2012)^27^ examined the Medicare costs of heart failure patients receiving care via video versus face-to-face, in the US setting. At 30 days, the average cost to Medicare for the telehealth patients was $4686 (SD $11,447) and face-to-face patients $4149 (SD $12,038). At 90 days, the average cost to Medicare for the telehealth patients was $7267 (SD $13,355) and face-to-face patients was $8048 (SD $15,118).

### Secondary outcome: patient satisfaction

Four trials reported on the patient satisfaction with care, however, data were not meta-analysable.

Augestad 2020^28^ reported that 59% of the face-to-face consultations and 41% of the telehealth consultations were rated as a ‘good overall experience’ by patients.

Campbell 2015 (the ESTEEM trial)^29^ reported patient satisfaction scores with care received on the day. Very similar percentages of patients responded that they were very satisfied (66% F2F, 65% telehealth); fairly satisfied (26% F2F, 25% telehealth); neutral (5% F2F, 5% telehealth); fairly dissatisfied (2% F2F, 3% telehealth); and very dissatisfied (1% F2F, 1% telehealth).

Gruffydd-Jones 2005^26^ reported that all of the patients in both the face-to-face and telehealth groups ‘were “satisfied” or “very satisfied” with the system of asthma care that they had received over the year.’

McKinstry 2002,^30^ measured the patient perception of the consultation using the Patient Enablement Instrument (PEI). Patient perceptions of the consultation were found to be similar in telehealth and face-to-face groups.

### Secondary outcome: care provider satisfaction

No trials reported on this outcome.

## Results comparison 2: telehealth via video vs. telehealth by telephone

### Results of the search

**Figure 8.**
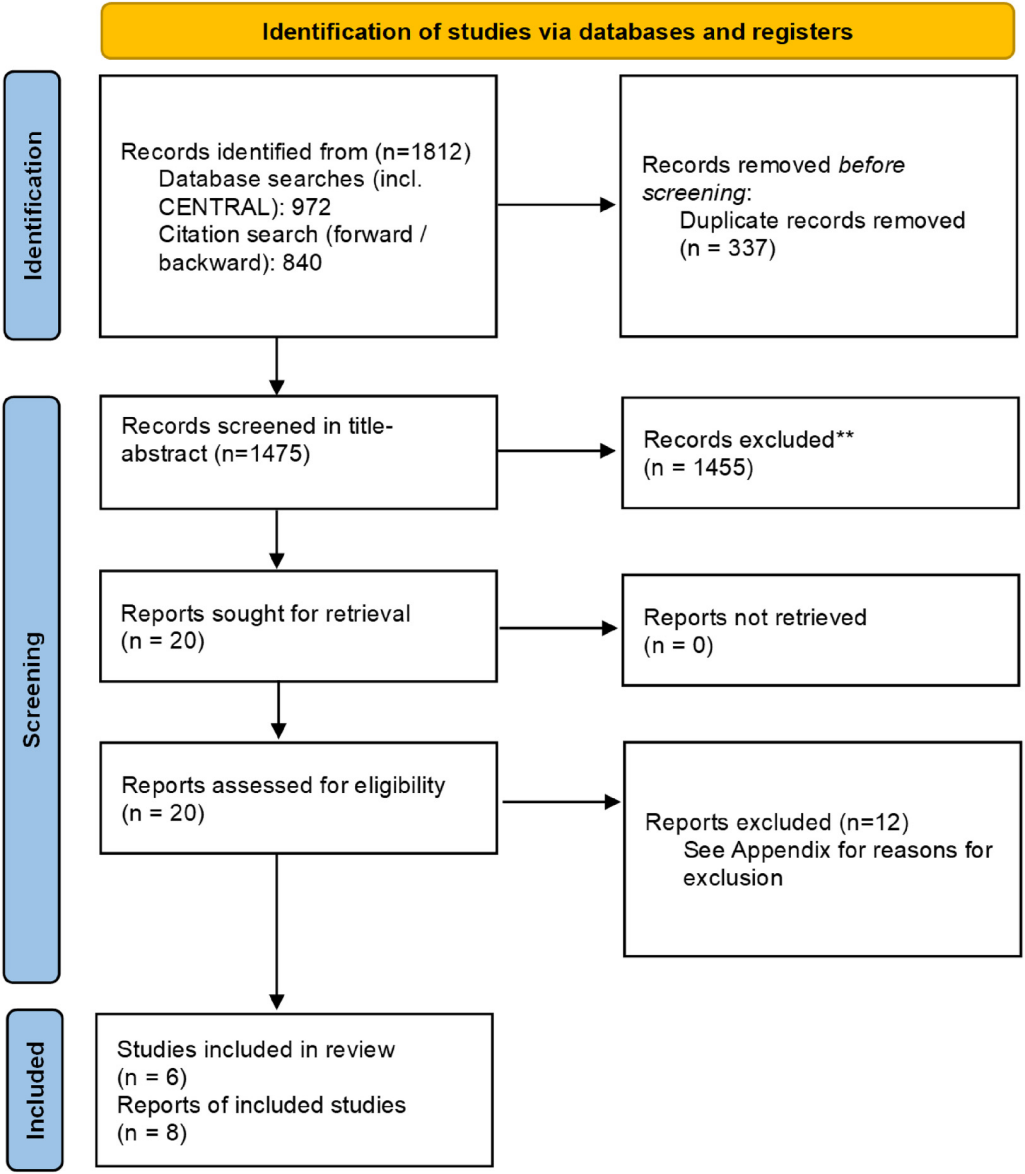
PRISMA flow diagram.

The searches yielded a total of 1812 references – 972 from database searches and 840 from forward and backward citation search. After deduplication, 1475 references were screened in title and abstract, and 1455 were excluded. We screened 20 references in full text; six trials (reported across eight references) were included (Figure 8).

### Characteristics of the included studies

Six randomised controlled trials were included (eight reports). Of those, four were individually randomised trials and two were cluster randomised trials (of those, one was a stepped wedge trial). The majority were conducted in the United States (four trials), with one trial each conducted in Australia and China. The oldest trial was conducted in 1975. Three trials were conducted with hospital outpatients, one with discharged hospital patients, one in nursing homes and one in primary care. Duration of follow-up ranged from 48 h to 12 months. For trials which reported individual patient numbers, the trial sizes were generally small, ranging from 37 to 148. All trials compared video (in one case, video via television) to telephone (Table 2).

**Table 2.** Characteristics of included studies (comparison 2: video vs telephone care).

Author, Year (Country)	RCT Design	Setting	Follow-up duration	Participants	Number of participants randomised N total (N Video group, N Phone group, N other groups if applicable)	Intervention type	Intervention: dose, duration, frequency	Comparator type	Comparator dose, duration, frequency	Sample size calculation (SS)? Powered (P)?
Jerant 2001 Jerant 2003 (^34,35^) USA	Parallel 3-arm	Hospital outpatient	Up to 180 days	Patients aged 40 or older admitted with a diagnosis of CHF	37(13 Video, 12 Phone, 12 UC*)	F2F baseline at 60 days + 11.7 (mean) visits by Video	Received video calls from a nurse and had access to nurse 8am to 5pm	F2F baseline at 60 days + 8.6 (mean) visits by Telephone	Received telephone calls, and had access to nurse 8 am to 5 pm	SS: Yes P: Yes
Li 2020(^48^) China	Parallel 2-arm	Hospital outpatient	Up to 3 months	Adult (18+) eligible stroke patients from a rehab hospital	120 (60 video; 60 phone)	F2F baseline visit + 2 visits Video	Baseline assessment at week of discharge followed by 2 mHealth app follow-up sessions by video (at 2 wks and 3 months); follow-up by research assistants with physiotherapy, occupational therapy or rehabilitation medicine background.	F2F baseline visit + 2 visits Telephone	Baseline assessment at week of discharge followed by 2 mHealth app follow-up sessions by telephone (at 2 weeks and 3 months)	SS: No P: No
Moore 1975(^36^) USA	Cluster 2-arm	3 remote health stations (Primary care)	7.5 months	Patients of 3 different remote health stations	Each day 2 of 3 remote health stations were randomly assigned to television; 3^rd^ station provided care by telephone	Baseline Video (via television)	Approx. 50-min consultation (baseline consultation by nurse with referral to GP if necessary)	Baseline Telephone by nurse, refer to GP if necessary	Approx. 40 min consultation	SS: No P: No
Phillips 2001(^49^) USA	Parallel 3-arm	Hospital outpatient	Up to 12 months	Patients 18 to 60 years old with a newly acquired spinal cord injury	111 (36 video, 36 phone, 39 UC*)	Video baseline + 5 sessions 1× a week + 2×in 1 month + regular care	Individual educational rehab sessions by video of 30–40 min with trial nurse 1/week for 5 weeks, then 1/every 2 weeks for 1 month in addition to regularly scheduled care	Telephone baseline + 5 sessions 1x/week + 2× in 1 month + regular care	Individual educational rehab sessions by telephone of 30–40 min with trial nurse 1/week for 5 weeks, then 1/every 2 weeks for 1 month in addition to regularly scheduled care	SS: Yes P: No
Sunner 2023(^33^) Australia	Stepped wedge cluster	Nursing home and ED	Up to 48 h	Patients in nursing homes	Total of 8 clusters (each cluster had 1 ED and 2 RACFs)	Nurse-led Video to ED at baseline + 1× phone call	ED ACE(9–4pm) with the addition of visual telehealth consultation (VTC); nurse-led	Nurse-led Telephone call to ED at baseline + 1 phone call	ED ACE access 9–4pm via phone only (no video)	SS: Yes P: Yes
Wakefield 2008 Wakefield 2009(^31,32^) USA	Parallel 3-arm	Discharged hospital patients	Up to 12 months	Patients admitted to hospital for heart failure	148 (52 Video; 47 Phone, 49 UC*)	F2F at baseline + 14×Video	Contacted 3 times by a nurse, by video the 1^st^ week, then 1× week for 11 weeks. Had plan of care. Could also contact nurse if needed	F2F at baseline + 14×Telephone	Contacted 3 times by telephone the 1st week, then 1× week for 11 weeks. Had plan of care. Could also contact nurse if needed	SS: Yes P: Yes

F2F: face-to-face; UC: usual care; TH: telehealth; RCT: Randomised controlled trial; ED: Emergency department; GP: general practitioner/general practice; RACF: residential aged care facility; NH: nursing home; COPD: chronic obstructive pulmonary disease; HF: heart failure; MNA: Mini Nutritional Assessment; ACE: Aged care emergency service. ^∗^F2F/UC groups are excluded from the present analyses; if the F2F and telehealth (either video or phone) groups were comparable and met the inclusion criteria, they are analysed in the TH vs F2F results section

### Assessment of risk of bias

#### Risk of Bias Tool-2 for parallel arm trials (n = 4 trials)

**Figure 9.**
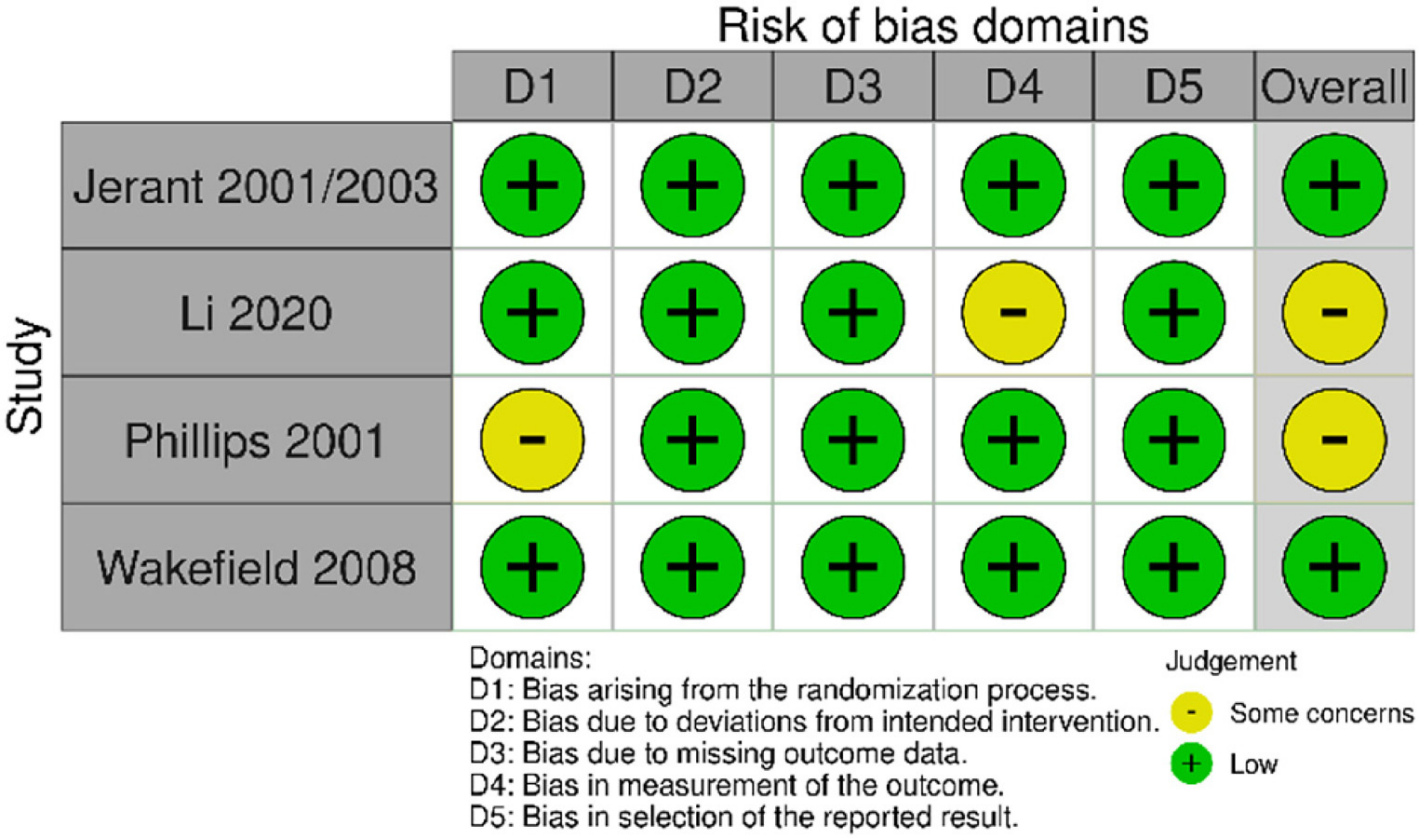
Risk of bias (parallel arm randomised trials).

Four included parallel arm trials were overall rated at either low risk of bias (two trials) or some concerns (two trials). The rating of some concerns was due to concerns about the measurement of the outcome in one trial, and due to randomisation process in one trial (Figure 9).

#### Risk of Bias Tool 2 for cluster randomised trials (n = 2)

**Figure 10.**
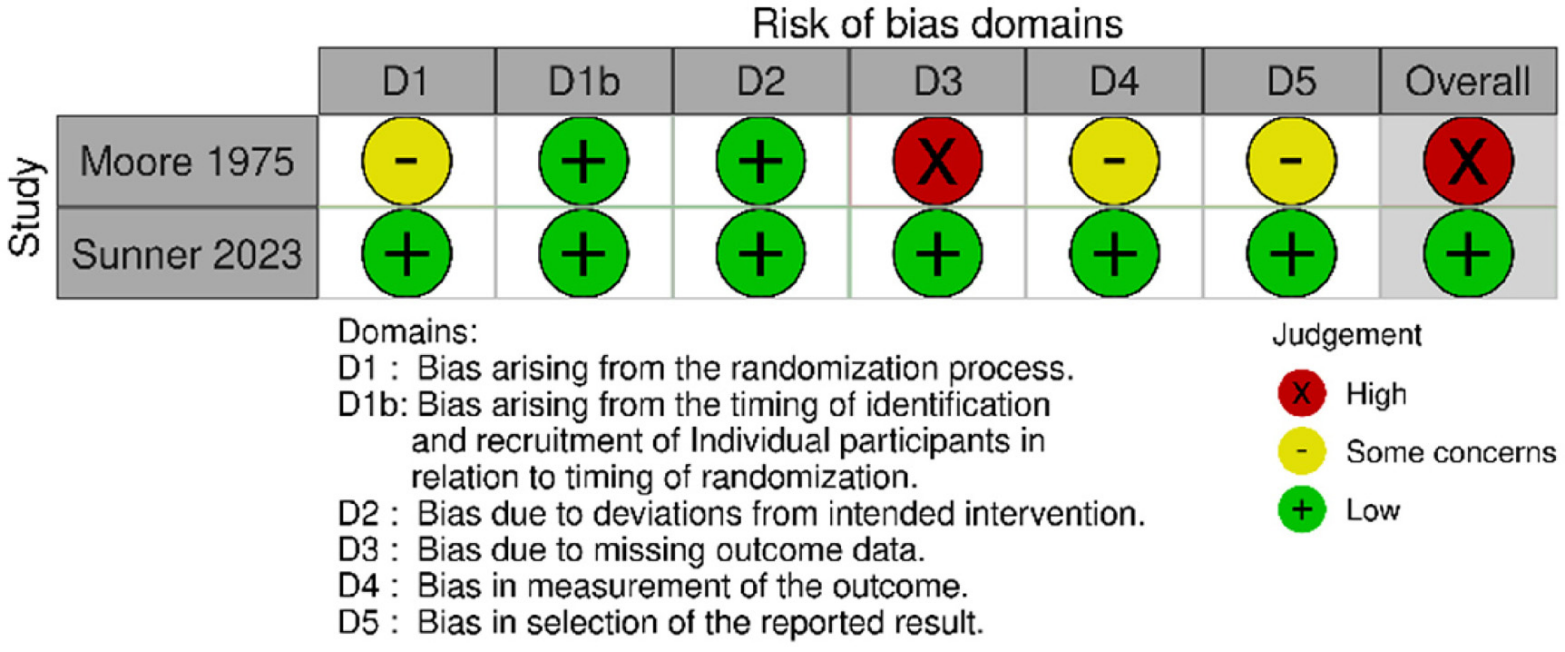
Risk of bias (cluster randomised studies).

Two cluster randomised trials were included. One trial was rated at an overall low risk of bias, owing to low risk of bias rating across all domains. Another trial was overall rated at high risk of bias, due to a high risk of bias rating for the missing outcome domain; it was also rated as some concerns in the randomisation, outcome measurement and selection of the reported result domains (Figure 10).

### Primary outcome: visits to the emergency department

Four trials reported on this outcome; three trials were meta-analysable.

**Figure 11.**
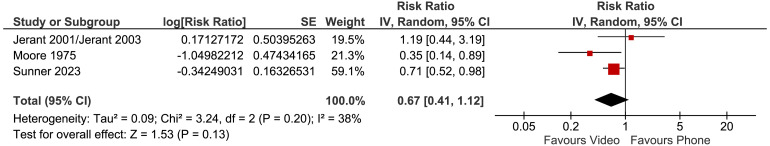
Video vs phone telehealth – impact on visits to the emergency department – random effects model (prespecified approach).

There was no significant difference between the video and phone groups for visits to the emergency department (three trials, RR 0.67, 95% CI 0.41 to 1.12, p = 0.13); heterogeneity of the pooled trials was low (I^2 ^= 38%) (Figure 11).

**Figure 12.**
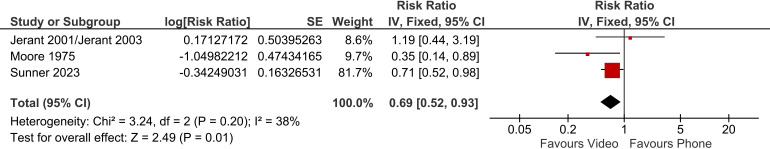
Video vs phone telehealth – impact on visits to the emergency department – fixed effects model (exploratory approach).

We conducted an exploratory analysis to test the impact of using the fixed effects model instead of the random effects model (as prespecified in the protocol). When the fixed effects model was applied, there *was* a significant difference, favouring video (three trials, RR 0.69, 95% CI 0.52 to 0.93, p = 0.01); heterogeneity of the pooled trials was low (I^2 ^= 38%). This finding needs to be interpreted with caution, however, due to the small number of included trials, and the exploratory (post-hoc) nature of the analysis (Figure 12).

One trial reported on this outcome but was not meta-analysable. The trial evaluated discharged American patients after hospitalisation for heart failure, found no significant difference in emergency department visits between the patients in the video and the phone groups.^31,32^

### Primary outcome: hospital admissions

**Figure 13.**
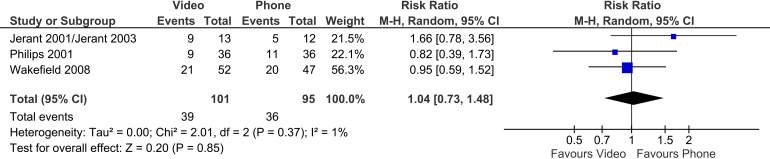
Video vs phone telehealth – impact on hospitalisations.

Three trials reported on hospitalisation outcome. There was no significant difference between the video group and the phone group in the number of hospitalisations (three trials, n = 196, RR 1.04, 95% CI 0.73 to 1.48, p = 0.85); heterogeneity between trials was very low (I^2 ^= 1%) (Figure 13).

### Secondary outcome: safety (including adverse events, deaths)

#### Three trials reported on safety outcomes

**Figure 14.**
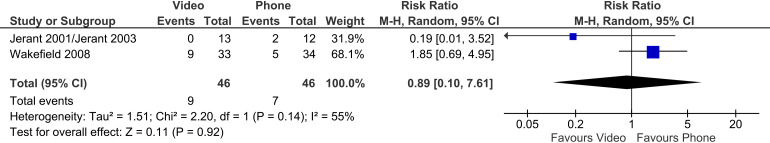
Video vs phone telehealth – impact on deaths.

Two meta-analysable trials reported the number of deaths, showing no significant difference between the video and the phone groups (two trials, 92 patients, RR 0.89, 95% CI 0.10 to 7.61, p = 0.92). Heterogeneity was moderate (I^2 ^= 55%) (Figure 14).

One trial reported on the presentations to the emergency department after video consultations to identify adverse events.^33^ Whilst telephone care was implemented, one aged care resident had an unplanned presentation to emergency within 48 h to identify adverse events. Whilst video care was implemented, two aged care residents had unplanned presentations (although neither had reported adverse events – one required referral for wound care follow-up; one required investigation for hip pain).

### Secondary outcome: costs

One trial^34,35^ evaluated the total care charges for patients with congestive heart failure, who were followed up for one year after discharge from a hospital in the United States. Median total care charges for the video group were $7487, and for the phone group $7117. Mean (standard deviation) total care charges for the video group were $29,701 (SD $49,219), and for the phone group $28,888 (SD $38,799). The differences between groups in total care charges were not significant.

Another, recently completed Australian trial, indicated that a future publication from the trial will present the cost consequence analysis.^33^

### Secondary outcome: patient satisfaction

**Figure 15.**
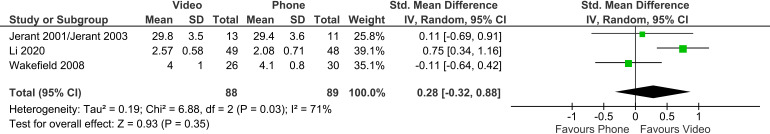
Video vs phone – patient satisfaction.

Four trials reported on patient satisfaction; three trials were meta-analysable. There was no significant difference, between patients accessing care via video and via telephone in patient satisfaction scores (three trials, n = 177, SMD 0.28, 95% CI −0.32 to 0.88, p = 0.35). Heterogeneity between trials was high (I^2 ^= 71%). (The level of heterogeneity is driven by the inclusion of the Li 2020 trial. Its removal from meta-analysis decreases the heterogeneity to 0%, whilst the difference between groups remains non-significant: SMD −0.04, 95% CI −0.48 to 0.40, p = 0.84, I^2 ^= 0%) (Figure 15).

One non-meta-analysable trial found that patients were generally satisfied with both video and phone consultations, and further reported that: ‘when asked if they would prefer to have seen the physician in person, less than 5% said that they would, with no difference between television and telephone users.’^36^

### Secondary outcome: care provider satisfaction

Two trials reported on care provider satisfaction, but reporting precluded meta-analyses.

One trial found that nurses and physicians had a similar, high level of satisfaction with video and phone consultations, with no strong preference for either modality. However, care providers seemed more likely to prefer video, when either visualisation of findings or patient teaching was required.^36^

One trial, which surveyed 44 care providers (majority were nurses), found that 38 of 44 respondents (88%) strongly agreed or agreed that the video teleconferencing was easy to set up, and 39/44 (91%) strongly agreed or agreed that it was easy to use. All respondents (44/44) agreed that video telehealth enhanced communications, and 97% felt that video telehealth provided a person-centred approach.^33^

## Discussion

In comparison 1, we identified 10 trials comparing telehealth care (five trials by telephone; four trials by video; one trial by both) to face-to-face care for transfers to the emergency departments. All trials were conducted in high-income countries, across a variety of settings, including: primary care (three trials); nursing home (one trial); hospital outpatient (two trials); hospital patients post-discharge (four trials). The quality of the evidence was good; six of the trials were rated low risk of bias overall, three were rated as some concerns and one overall high risk of bias. Overall, these trials found no difference between telehealth and face-to-face consultation for transfers to ED and hospitalisations.

In comparison 2, we found six trials comparing live telehealth via video to live telehealth via phone. Most trials were US-based (n = 4), but involved a variety of participants, including hospital outpatients, discharged patients, nursing home residents and primary care patients. The quality of the evidence was mixed, with half of the trials (n = 3) rated at low risk of bias. Overall, there was no difference between the video and telephone groups for: visits to the emergency department, hospitalisations, safety (deaths), costs and patient and care provider satisfaction.

We identified several knowledge gaps in the existing evidence. First, the number of trials is limited to 10 (for the telehealth via video or phone to face to face comparison) and six (for the video to phone comparison). The limited volume of trial evidence precluded us from conducting planned subgroup analyses by health condition. The volume of the existing evidence would also be insufficient to analyse whether the use of telehealth has different impacts on escalation to emergency departments or on hospital admission by the different patient groups, i.e., primary care patients, hospital discharged or outpatients and nursing home patients. However, this may change as the volume of evidence on live telehealth increases with its greater integration into health systems post-COVID. Second, the existing evidence is predominantly from higher income countries. Fifteen of the included studies were from Europe, United States or Australia; only one trial was from China. This shows important gaps in the existing evidence from Asia, Africa and South America. Finally, the evidence for the care provider satisfaction outcome is limited to two studies, both in the video to phone telehealth comparison. As healthcare providers’ dissatisfaction with it may limit its adoption,^37^ it is important to collect this outcome as part of future telehealth trials.

Our findings are consistent with other systematic reviews, which showed that there are generally no differences in outcomes for live telehealth versus face to face care,^12,38–40^ or for live telehealth via video versus by phone,^41^ for a broad range of patient groups and health conditions. They are also consistent with the findings of an analysis of over 6900 electronic health records of discharged patients with heart failure, which showed that a 30-day hospital readmission rate was similar for patients receiving telemedicine visits (15%) and those receiving in-person visits (14%).^42^ On the other hand, a previous scoping review, examining whether telehealth influences the decision to transfer the residents of aged care facilities specifically to the emergency departments,^20^ found mixed results. This may be due to the fact that it included predominantly non-randomised studies, and did not meta-analyse the studies. There is systematic review evidence, that telehealth decreases the number of hospitalisations, and the number of all-cause hospital days for patients across a broad range of conditions.^43^ However, the difference in finding to our review may be due to the comparison (to usual care) and the broader inclusion of telehealth types (including both synchronous and asynchronous telehealth).

The strengths of our review include a comprehensive search of databases, trial registries and a forward and backward search on the included studies to identify further evidence. The synthesised trials also evaluated outcomes for range of patient groups, including from primary care, hospital discharged or outpatients and nursing home patients. However, one of its limitations is the inclusion of only the evidence from randomised controlled trials. By excluding evidence from observational studies, we limited the total volume of evidence that may have otherwise been includable. This choice was driven by the consideration that biases are likely greater for non-randomised study designs when addressing intervention questions.^22^ The review also excluded studies that provided asynchronous telehealth (such as via mobile apps, online platforms, text, email support and store and forward technologies, for example), which also decreased the volume of includable evidence. Finally, as noted above, because all but one of the included studies were from Australia, North America or Europe, the generalisability of the present findings is limited outside of those settings.

Telehealth care – delivered by telephone or by video – may be an appropriate alternative to face-to-face provision of care, as it does not increase the likelihood of escalation of care to the emergency department for patients in primary care, hospital outpatients, post-discharge patients or residents in aged care. This is a relevant consideration particularly for those clinicians who provide care to those patient groups, and for the healthcare systems which utilise separate funding mechanisms for the funding of primary and hospital care.
